# Techno-economic assessment of power generation potential from floating solar photovoltaic systems in Bangladesh

**DOI:** 10.1016/j.heliyon.2023.e16785

**Published:** 2023-05-29

**Authors:** Md Fatin Ishraq Faruqui, Atik Jawad, Nahid-Al- Masood

**Affiliations:** a Department of Electrical and Electronic Engineering, Bangladesh University of Engineering and Technology, Dhaka 1205, Bangladesh; b Department of Electrical and Electronic Engineering, University of Liberal Arts Bangladesh, Dhaka 1207, Bangladesh

**Keywords:** Floating PV, Solar power potential, Inland water infrastructures, Bangladesh, NPV, IRR

## Abstract

Floating Solar Photovoltaic (FPV) plants, also known as floatovoltaic plants are showing great potential in the renewable energy sector all around the world. They can contribute to the national grid and provide support to the existing hydropower plants. Moreover, they positively impact the environment by reducing evaporation and improving aquatic lives simultaneously. Despite a decade of research, there has been no study on the technical potential of FPV plants on a riverine country like Bangladesh. In Bangladesh, there are several water infrastructures to accommodate FPV plants. In addition, a considerable amount of solar irradiation is available throughout the year because of the country's geographical location, making FPVs a significantly viable option for generating electricity. To this end, this study provides the first technical potential and economic feasibility assessment of some of the important water bodies of Bangladesh. The technical potential study is performed with the help of solar PVGIS and focuses on the contribution these plants can make to the national grid. The economic viability assessment simulations are done in System Advisory Model (SAM). Moreover, a thorough comparison between FPVs and inland solar plants is also conducted. The results show that after the installation, even with a conservative approach, FPV plants will be able to meet 1.1% of the demand of the capital Dhaka, the city with one of the highest population densities. In addition, FPV installation at Kaptai lake, which already has an existing hydropower plant, can meet up to 7% of the demand of the port city Chattogram. Moreover, economic parameters NPV, IRR and LCOE all indicate the projects are profitable and can be deployed in large-scale. This study will open doors to further research into the FPV potential of Bangladesh and help implement FPVs to meet the renewable energy goals of the country.

## Nomenclature

${Α}_{lake}$: Lake coverage (m^2^)
${Α}_{panel,c}$: Solar panel coverage area (m^2^)
δ: Tilt angle (degree)
${Α}_{panel}$: Solar panel area (m^2^)
$W_{p}$: Installed peak power (kWp)
$GHI_{p}$: Peak global horizontal irradiation (kWp/m^2^)
η: Efficiency at Standard Test Conditions (STC) (%)
C: Capacity factor (%)
$E_{p}$: Generated energy during analysing time period (kWh)
T: Time period (h)
κ: AC-DC ratio
$W_{inv}$: Inverter rating (W)
$W_{module}$: Module rating (W)
$N_{T}$: Total number of required modules
$V_{\max}$: Inverter maximum operating voltage (V)
$V_{\min}$: Inverter minimum operating voltage (V)
$V_{oc}$: Module open circuit voltage (V)
$N_{s,\max}$: Maximum modules per string
$N_{s,\min}$: Minimum modules per string
$F_{n}$: Net cash flow in year n
d: Annual discount rate (%)
N: Time period
NPV: Net Present Value ($)
IRR: Internal rate of return (%)
LCOE: Levelized Cost of Energy
$Q_{n}$: Electricity supplied by the system to the grid in year n (kWh)
$C_{n}$: Annual project cost ($)

## 1 Introduction

Solar photovoltaic technology has been one of the main interests of the researchers in the renewable energy domain for the last couple of decades. Renewable energy is the solution to obtain a sustainable development [1]. The renewable energy sources are the way to emancipate the world from depletion of ozone layer and other environmental problems associated with it. Among the sources, solar plants are among the best in case of output generation especially in Asia region along with hydro power and geothermal energy [2]. Though overland solar photovoltaic (PV) plants have their own merits, floating photovoltaic (FPV) plants have clear advantages since it can increase energy efficiency, increase output generation due to lower operating temperature, improve health of aquatic animals, control algae and other harmful plants growth, and reduce water loss due to evaporation [3].

### 1.1 Literature review

A detailed study by Spencer et al. assessed the technical potential of FPV plants in United States [4]. In the study, 27% of the suitable water bodies were taken in consideration which could produce 10% of the total generation of the United States. This was the first national level analysis for a whole country and paved the way for other researchers to emulate the same for their respective countries. Choi et al. analyzed the already installed FPV plants and evaluated their efficiency [5]. Moreover, effect of wind speed and waves on FPV plants were taken into consideration. It showed that the FPV plants are 11% more efficient than their inland counterparts. But the economic analysis was left out of this study which could not demonstrate the profitability. Research by Farfan et al. introduced the concept of combining FPV plants with hydro power plants [6]. The study showed the 6.3% more water availability and additional 142.5 TWh generation of electricity. But the study did not take economic analysis into consideration. Moreover, the social and legal barriers were also not part of this research. Clemons et al. evaluated the generation potential and economic profitability as a case study of Thailand [7]. But it left out the water rights or legal obligations which the concerned authority would face. The social and legal obstacles faced while installing the FPV plants are rare and can be a scope for future research. Mittal et al. in a case study, showed Kota barrage could produce 18,38,519 kWh energy per year and Kishore Sagar Lake could produce 18,58,959 kWh per year [8]. Additionally, 37 million litres of water could be saved from this project. However, this was a case study which was limited to two water bodies in Rajasthan, India. Dorenkamper et al. evaluated a rather untouched aspect of the study which is the cooling effect of the FPV panels [9]. The research was conducted in two separate climate conditions and both cases showed better energy yield with respect to overland PV plants. But the economic feasibility was out of consideration for this research as well. Kichou et al. did simulation-based research which demonstrated the FPV plants to be 3% better in terms of performance [10]. The study also included the economic profitability as well. All the mentioned studies demonstrated a distinct performance improvement in case of FPV plants over the inland ones. Tina et al. analyzed the performance of tracking panels for FPV plants and showed 3% better gain for Anapo dam in Sicily and 4% for Aar in Germany [11]. The focus of this study was limited to the addition of tracking panels, economic aspect of the proposed plants was out of context for this study. Rahman et al. evaluated the potential of solar lanes besides Kaptai Lake in Bangladesh [12]. The study showed the technical potential of the proposed plants but did not mention the feasibility of this study. Moreover, the study did not take any other water bodies into consideration. Considering all the conducted literature review, a concise summary regarding floatovoaltaic studies is given in Table 1 for convenience.

**Table 1:** Literature review.

Sr. No.	Author	Study	Major Findings	Research Gaps
1	Spencer et al. [4]	Technical potential in the continental USA.	27% of the number and 10% of the total area of the water bodies could provide 10% of the national generation in USA.	The study is approximation based and depends on the specific dataset on which the analysis is performed. Moreover, the study does not discuss the comparison of the FPV plants with their land-based counterparts.
2	Choi [5]	FPV power generation analysis considering environmental impact.	FPV installations are 11% better than their inland counterparts. Effect of environment on generation efficiency is also discussed.	Economic analysis not provided. No discussions done regarding the profitability of the project.
3	Farfan et al. [6]	Combining hydro power plants (HPP) with FPV plants.	Combining FPV and HPP increases water availability by 6.3% and adds approximately 142.5 TWh generation to HPP generation.	The study demonstrates only the HPP-FPV combination aspect, and economic and socio-environmental barriers are not discussed.
4	Clemons et al. [7]	Feasibility study of FPV plants in Thailand.	Thorough economic analysis is done on 21 water reservoirs of Thailand and shown that levelized cost of energy and payback period is lesser than the overland ones.	However, this study also could not demonstrate the social and legal barriers that FPVs face. Social impacts of FPV and perception of the general people is rare throughout the world.
5	Mittal et al. [8]	Feasibility of FPV plants in Rajasthan in India	The studies show that the FPV installed can save up to 37 million of water and reduce CO_2_ emission by 1714 tonnes per year.	This was a case study which took only four lakes of Rajasthan into account and left the other lakes out of consideration.
6	Dorenkamper et al. [9]	Cooling effect of FPVs in two different climates.	The study shows 3% better energy yield in Netherlands and 6% better in Singapore with respect to the overland ones.	The economic parameters and the barriers faced due to the installation of these FPV plants were out of consideration in this study.
7	Kichou et al. [10]	Simulation based analysis of FPV plants in Czechia.	The study shows 3% better performance than the terrestrial ones. It also introduced the tracking panels in the FPV plants which yields 20% more energy.	The contribution of the FPVs to the respective national grid was out of consideration of this study.
8	Tina et al. [11]	Energy performance evaluation of tracking FPV.	It shows gains over 3% in Sicily and 4% in Germany. This study also shows the gain achieved due to the cooling of the modules.	Economic analysis and contribution to the grid are out of scope of these studies.
9	Rahman et al. [12]	Solar lanes and technical assessment of Kaptai lake in Bangladesh.	The study shows that 13% of the total electricity generation can be generated through solar lanes and a portion of Kaptai lake.	The economic feasibility is out of scope for this research. Moreover, among the water bodies, only Kaptai lake is analyzed, which does not represent the scenario of the whole country.

At present, there are 4 large scale solar power plants existing in Bangladesh: Teknaf Solar Park (28 MW), Sutiakhali (50 MW), Sunamganj Solar Park (32 MW) and Mymensingh Solar Park (40 MW) [13]. On the other hand, there is only one floating solar PV plant in the country. This plant in Mongla only has a capacity of 10 KW [14]. The Asian Development Bank (ADB) published three (3) feasibility reports on Barapukuria lakes, Bukbhara Baor and Joydia Baor in this regard [15]. The government aims to generate 40% of the electricity through renewables by 2041 [14]. Therefore, to meet the target of generation through renewables, large scale installation of solar FPV plants in Bangladesh is inevitable and requires much attention.

### 1.2 Research gaps and specific contribution

The research of FPV plants installation and their generation capacity have been conducted all over the world for quite some time now. But this is a comparatively new prospect in South-East Asia and the conducted research are still in preliminary stage with very few practical implementations. Considering all the discussed literature in the previous subsection, the crucial research gaps can be identified as follows:

• FPV studies are in progression in Bangladesh and few research have been conducted in this aspect, but those did not investigate how FPVs can assist the national grid. Furthermore, the reports do not represent the entire scenario of Bangladesh's FPV potential.
• Some researchers have highlighted on the possibilities of Kaptai Lake as a location for an FPV plant in Bangladesh. However, there are other major water bodies that can be utilized for FPV installation. To deploy FPV plants, a thorough performance evaluation and feasibility study are required in such locations.
• One of the most important factors to consider when adopting a new plant technology is the expenditure. Existing FPV-related research in Bangladesh does not address the economic viability of the proposed FPV plants.
• To move from inland solar plants to FPV plants or to install alongside the inland ones, a thorough comparative analysis is needed. However, none of the available works offer a comparative study from the viewpoint of Bangladeshi water bodies.

Taking all the research gaps into account, the purpose of this study is to unlock the generation potential and economic feasibility of FPV plants installed in Bangladesh based on some selected water bodies. In addition, the study provides a thorough comparative analysis of the inland PV plants and the proposed FPV plants based on technical, economic, and environmental aspects. The paper's main contribution is outlined in the paragraphs that follow.

• The research done earlier lacked a comprehensive analysis of the potential of FPV plants. Moreover, an economic analysis is always necessary to assess the practical implementation. Therefore, a systematic methodology is proposed to comprehensively assess the generation potential and economic feasibility of deploying FPV plants in the water reservoirs. The proposed method is generalized and can be applied to any potential FPV plant evaluation.
• The approach for FPV plant assessment is utilized on seven artificial lakes across different regions of Bangladesh. Each plant's viability is extensively assessed in terms of power generation, economic impact, and contribution to the national grid.
• A comparative analysis of inland PV plants and proposed FPV plants is provided based on generation ability, economic viability, cost variables, and environmental considerations.

The remainder of the paper is structured as follows. Section 2 describes the methodology and the approaches taken to evaluate the potential and viability of the FPV plants. Moreover, a flowchart of the study is also present in this section for convenience. The third section deals with the results associated with this study and analyze various aspects of the results such as the generation potential of the plants and economic viability. The next section demonstrates a comparison between the inland PV plants with the proposed solar FPV plants. In Section 5, a brief discussion on the limitations of the work is given. Finally, in the last section, we provide a succinct summary of our findings and insights.

## 2 Methodology

**Fig. 1:**
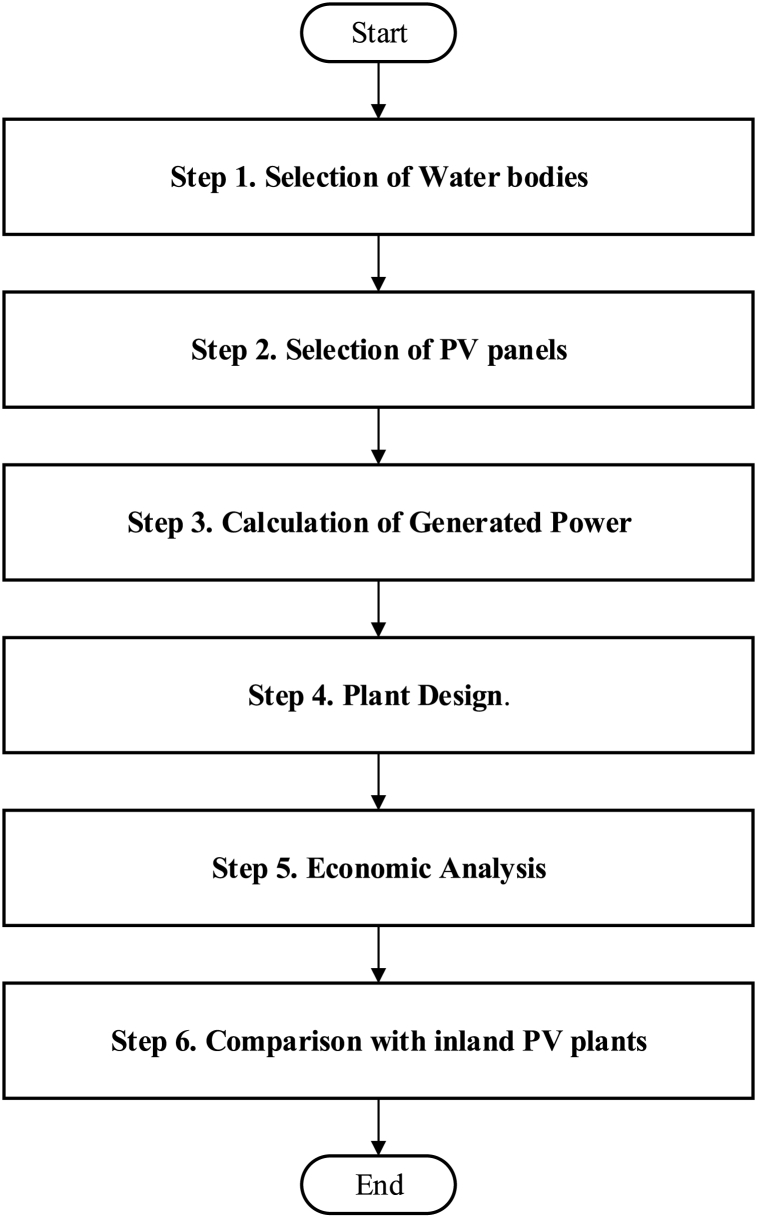
Flowchart of the proposed study.

This section presents a methodology to determine the generation potential and economic feasibility of the selected waterbodies. Moreover, designing of plant components are also provided in this section along with the selection of appropriate solar panels to be installed. The workflow of this study is shown in Fig. 1.

In the following subsections, every step of the methodology is described in detail.

### 2.1 Selection of water bodies

The primary requirement for the installation of FPVs is to identify suitable water bodies. In this study, seven lakes were selected from different regions: three from the capital Dhaka, one from the port city Chattogram and other three from different regions of the country. All the lakes were artificial, as it should be for FPV installation. This is because the man-made water reservoirs are generally stagnant and detached from the river or the ocean which makes them perfect for FPV installation [16]. Note that, the water bodies selected for the study do not include all the man-made water bodies in Bangladesh. But as this is a case study, the water bodies representing the whole country with high potential are selected. The three lakes of Dhaka (Dhanmondi Lake, Gulshan Lake and Hatirjheel lake) were chosen as these are the only man-made water bodies in the capital. Three of them (Joydia Baor, Bukhbhara Haor, Barapukuria lake) have been recommended by Asian Development Bank (ADB) and so those were also included in this study [15]. Kaptai lake is chosen because it has a Hydro power plant attached. Table 2 summarizes the reasons for selecting the specific water bodies along with their location. Location of the lakes along with their specific latitudes and longitudes throughout the country is shown in appendix for convenience. The appendix also includes the location of the lakes throughout the whole country and in their specific districts in separate figures.

**Table 2:** Selection of water bodies.

Lake	District	Selection remarks
Dhanmondi Lake	Dhaka	This lake is one of the few man-made reservoirs in the capital. The lake harbors residential as well as commercial infrastructures. Though a small portion of the lake is used for entertainment purpose such as boat riding, most of the area of this water body is unused which makes it a suitable choice of FPV plant installation.
Hatirjheel Lake	Dhaka	Hatirjheel lake also has boat riding facilities for entertainment but then again most of it remains unused even today. The lake is surrounded by important commercial buildings. The proposed FPV plants can meet the demand of these infrastructures and some of the adjacent area.
Gulshan Lake	Dhaka	Gulshan lake is situated in a very busy location where the shopping malls are located. The lake is stagnant and is unused for many years. These make the lake suitable for FPV implementation.
Joydia Baor, Bukhbhara Haor and Barapukuria lake	Dinajpur, Jessore, and Jhenaidah	These three lakes were recommended by Asian Development bank (ADB) [15]. The water bodies are stagnant and unused for a long time which makes these suitable for FPV installation. Moreover, these lakes harbour conventional generation grid, which makes them suitable for the proposed study.
Kaptai Lake	Chattogram	Kaptai lake is one of the largest lakes in Bangladesh. A portion of it is used for Hydro power plant, but the rest of it is mostly unused. HPP attachment helps the cause of FPV installation which is already done in many countries. These reasons make this lake suitable for the proposed study.

### 2.2 PV panel characteristics

To determine the power generation by the installed floating solar panels, the following PV panel specifications are the standard ones which are used in PV panel studies [17]. The specifications for this study are shown in Table 3.

**Table 3:** PV panel specifications.

Site info	
PV technology	Crystalline Silicon
Mounting	Fixed
Mounting options	Free-standing
Tilt	11°
Azimuth	0
**System loss**	
Soiling	2%
Shading	3%
Snow	0%
Mismatch	2%
Wiring	2%
Connections	1%
Light-Induced Degradation	2%
Nameplate Rating	1%
Age	0%
Availability	3%
Total	14%
**Panel dimensions**	
Length	1956 mm
Width	992 mm
Height	40 mm

### 2.3 Calculation of generated power

After selecting the aforementioned water bodies, suitable area of installing FPVs were carefully identified. The whole area of the lakes should not be used for couple of reasons. Lakes can be a source of amusement, have active route of water transportation, or be home to hydroelectric power plants, as in the case of Kaptai lake at Chattogram. In some cases, the livelihood of the people around the basin depends on the lakes such as the fishermen. Therefore, a conservative approach was taken by installing virtual solar panel on Google Earth Pro and then the area was calculated in percentage of the whole area of the lakes [18].

Next, to assess the solar power potential at the intended locations, solar irradiation profile was obtained from global solar irradiation database, PVGIS-SARAH, which has the broadest coverage of irradiation data [19]. In order to determine peak power of the solar panels, the total area and efficiency of the solar panels are required. Solar panel coverage area includes the floating system, the mooring system, floated, buoyancy anchor, underwater cable, etc. It can be determined using Eq. (1).

(1) $${Α}_{panel,c}=61\%\times {Α}_{panel}$$

Where, ${Α}_{panel}$ is the lake coverage (m^2^), and ${Α}_{panel,c}$ is the solar panel coverage area (m^2^).

Afterwards, the area of the solar panels area can be found from Eq. (2) [20].

(2) $${Α}_{panel}=\frac{{Α}_{panel,c}}{\cos\left(\delta \right)}$$

Where, δ is the tilt angle in degrees. Generally, the tilt angle of solar panels depends on the part of the world where it is located. For Bangladesh, the tilt angle for floating solar panels is considered to be 11° in case of horizontal plane [4]. The efficiency of a solar panel using polycrystalline cells was considered to be 17% [21]. Usually, the polycrystalline material is used mostly in the world for solar panels because it is the cheapest, and environmentally friendly. Therefore, installed peak power can be calculated from Eq. (3) [20].

(3) $$W_{p}=GHI_{p}\times A_{panel}\times \eta$$

Where, $W_{p}$ is the installed peak power (kWp), $GHI_{p}$ is the peak global horizontal irradiation (kWp/m^2^), and η is the panel efficiency (%) at Standard Test Conditions (STC). $GHI_{p}$ (Global Horizontal Insolation) was assumed to be 1 (kWp/ $m^{2}$). Generation capability of the FPV plant in different months and the average yearly generation were calculated using PVGIS, which is a free solar photovoltaic energy calculator for stand-alone or grid-connected solar PV plants [22]. Previously determined installed peak PV power, and tilt angle of the solar panels are given as input to PVGIS.

Next, capacity factor, C, on monthly basis was determined using Eq. (4).

(4) $$C=\frac{E_{P}}{W_{P}\times T}\times 100$$

**Fig. 2:**
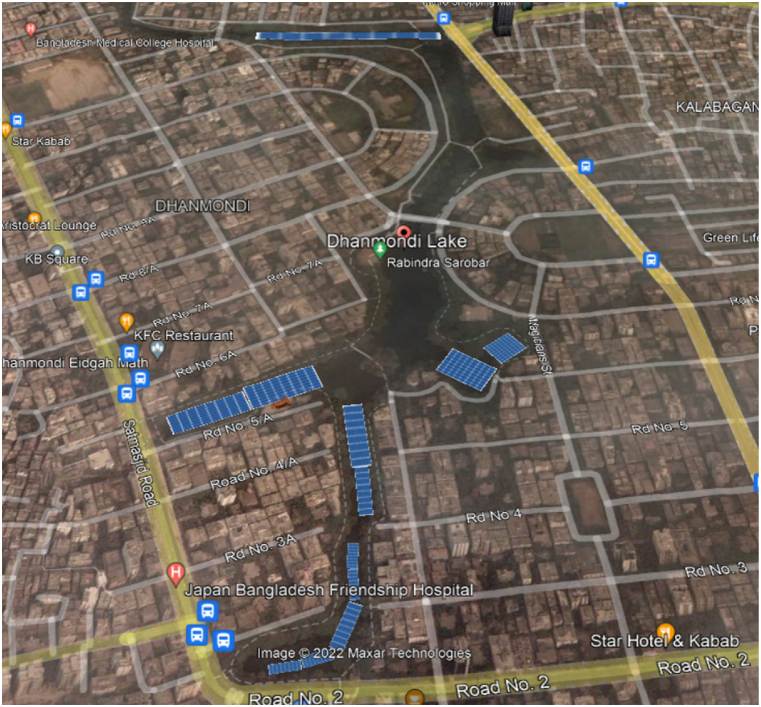
Proposed FPV installation at Dhanmondi Lake, Dhaka.

Where, $E_{p}$ is the generated energy during analysing time period (kWh), and T is the time period (hours). A typical floating PV plant can be seen from Fig. 2. It generally consists of solar panels along with mooring devices. The underwater cables are connected with the inverters installed on land which then transmissions power through the network. Moreover, a map with solar PV installations in one of the lakes of this study is shown in Fig. 2 as an example [5].

In order to compare the contribution of the generated electricity to the national grid, monthly average demand information was collected from BPDB (Bangladesh Power Development Board) website [23]. In general, the candidate days were 15th day of the month, unless it was a weekend; in that case nearest weekday was selected. Then the average peak demand was compared with previously determined installed power of the FPV plant in order to obtain the percentage of aid to the national grid.

### 2.4 Plant design

To practically realize the results found from PVGIS, a design for the proposed FPV farm is proposed at this stage. At first, the inverter with proper rating must be selected as per Eq. (5).

(5) $$W_{inv}=\frac{W_{P}}{\kappa }$$

Where, $W_{inv}$ is the inverter rating (W), and κ is the AC-DC ratio. Afterwards, a module rating is to be assumed in order to get the number of total modules required, which is calculated as per Eq. (6).

(6) $$N_{T}=\frac{W_{P}}{W_{module}}$$

Where, $W_{module}$ is the module rating (W), and $N_{T}$ is the total number of required modules. For that reason, an initial assumption is to be made about the rating of the solar modules, which can later be modified to give a better performance.

Next, the string configuration of the solar panels is to be determined. In order to calculate that, the inverter maximum and minimum voltage, and module open circuit voltage (VOC) are to be known.

(7) $$N_{s,\max}=\frac{V_{\max}}{V_{oc}}$$

(8) $$N_{s,\min}=\frac{V_{\min}}{V_{oc}}$$

Where, $V_{\max}$, $V_{\min}$, $V_{oc}\text{,}$ $N_{s,\max}$, and $N_{s,\min}$ are inverter maximum operating voltage (V), inverter minimum operating voltage (V), module open circuit voltage (V), maximum modules per string, and minimum modules per string respectively. Any number of modules per string can be picked from this range of maximum and minimum number of modules as given by Eqs. (7), (8).

### 2.5 Economic analysis

Based on the design proposed in the previous subsection, an economic analysis is carried out to evaluate the implementation feasibility of the plant. Economic viability is determined based on three (3) indices, which are:

1. **Net Present Value (NPV):** NPV is one of the important measures to evaluate a project's economic feasibility. NPV is in general a comparison between cash inflows and cash outflows and is defined as per Eq. (9).

(9) $$NPV=\sum_{n=0}^{N}\frac{F_{n}}{{\left(1+d\right)}^{n}}$$

Where, NPV is the Net present value, F_n_ is the net cash flow in year n, N is the time period in which the analysis took place, and d is the annual discount rate.

When NPV>0, it means after the analysis period, the revenues (cash inflow) exceed the cost which indicates that the project is economically feasible. But only from NPV, one cannot ensure a project's viability; for that, other economic parameters must be evaluated [24].

2. **Internal Rate of Return (IRR):** IRR is the measure of discount rate at which NPV becomes zero at the end of the analysis period. IRR is defined as per Eq. (10):

(10) $$NPV=\sum_{n=0}^{N}\frac{F_{n}}{{\left(1+IRR\right)}^{n}}=0$$

Generally, the higher the IRR, the better. So, when the IRR of a project exceeds the cost of capital and investments, then the project can be accepted [24].

**3. Levelized Cost of Energy (LCOE):** The levelized cost of energy is the total cost expressed in cents for per kW-hour of electricity delivered by the system. LCOE is defined as per Eq. (11):

(11) $$\sum_{n=1}^{N}\frac{Q_{n}\times LCOE}{{\left(1+d\right)}^{n}}=\sum_{n=0}^{N}\frac{C_{n}}{{\left(1+d\right)}^{n}}$$

Where, Q_n_ denotes the electricity supplied by the system to the grid in year n, N is the analysis period, C_n_ is annual project cost, and d is the discount rate.

When LCOE is greater than market price, the project should be accepted provided that other parameters also indicate positive economic effects of the project [25].

### 2.6 Comparison with inland PV plants

Based on electricity generation and economic feasibility calculated from above mentioned steps, the inland PV plants vary from the proposed FPV plants. Moreover, the plants also vary in terms of environmental aspects as well as installation cost factors.

## 3 Results

The purpose of this section to assess the generation potential and economic feasibility of the proposed FPV plants following the mentioned methodology. The generation subsection is further categorized into Dhaka lakes, non-Dhaka lakes and Kaptai lake. Next, an economic evaluation and profitability of the plants are demonstrated in the subsequent subsections.

### 3.1 Solar power generation and capacity factor

The generation of the lakes was calculated by on the basis of the results derive from PVGIS irradiance values.

#### 3.1.1 Dhaka lakes

**Fig. 3:**
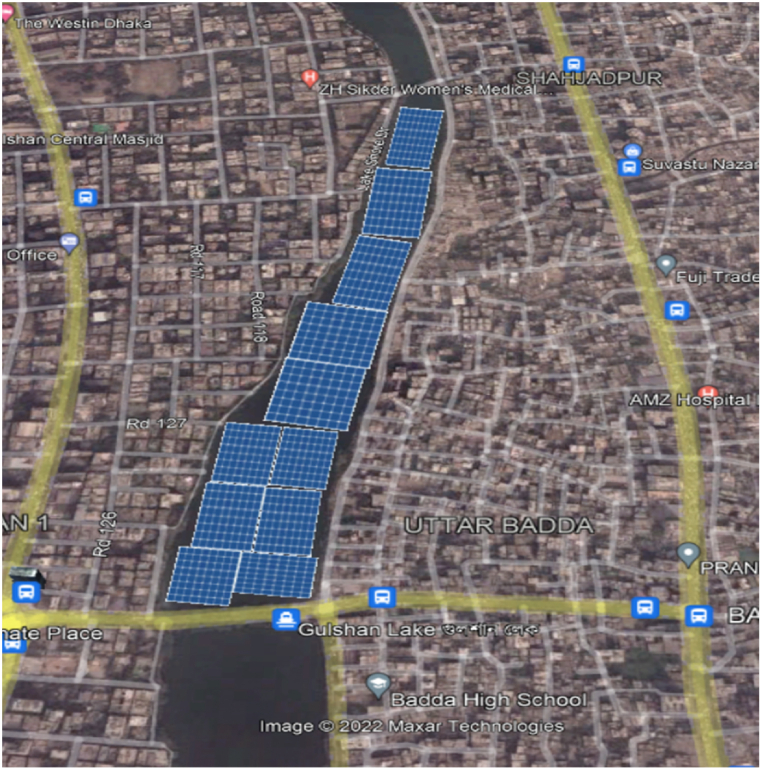
Proposed FPV installation at Gulshan Lake, Dhaka.

**Fig. 4:**
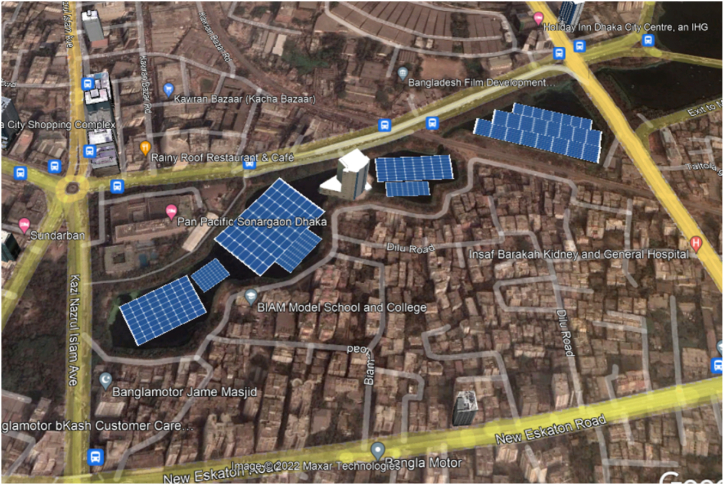
Proposed FPV installation at Hatirjheel Lake, Dhaka.

Dhaka, the capital of the country, consumes 46% of the total demand of Bangladesh [26]. Therefore, solar power generation can help taking the load off the main grid. In this study, three (3) lakes were considered for potential floating PV installation site, namely - Dhanmondi lake, Gulshan lake and Hatirjheel lake (Fig. 2, Fig. 3, Fig. 4).

As it is one of the densest megacities in the world with 23,234 people per square km [27], conservative approach was taken to finalize the location of potential floatovoltaic plants. Dhanmondi and Hatirjheel lakes have the facilities of water taxi and recreational boat riding. Gulshan lake has diplomatic buildings i.e., foreign embassies on its basin. Therefore, while calculating the coverage of floating PVs, it was taken into careful consideration that there was no impairment to the usual day-to-day activities of the nearby areas, and to the people residing on those locations. The areas were calculated drawing mini solar FPV panels on Google Earth pro. All the calculated areas were around 5% of the total surface area of the lake. Therefore, 5% of the total surface area was chosen as reference point for this study. Study on the floating PV potential for 10% and 15% of the total area of the lakes were also carried out. However, it was decided not to exceed 15% of the area for the sake of maintaining conservative approach.

**Fig. 5:**
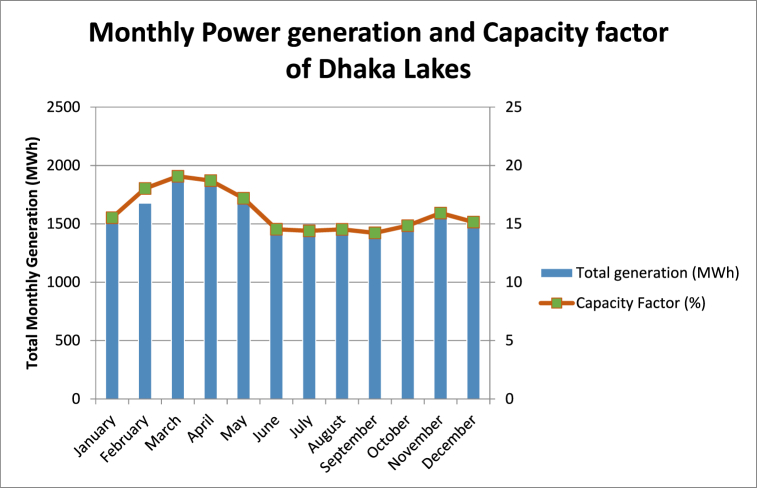
Monthly power generation and capacity factors of FPV installations at selected lakes of Dhaka.

Fig. 5 shows the monthly power generation and the respective capacity factors for the installed floating PV plants on the lakes in Dhaka. Capacity factors were determined using Eq. (4).

**Fig. 6:**
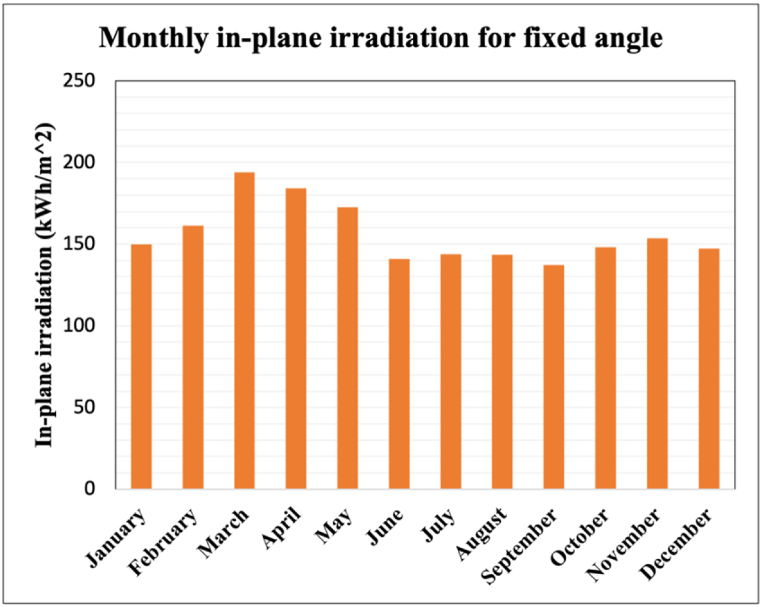
Monthly in-plane irradiation in Bangladesh.

It is evident from Fig. 5 that March and April have the highest potential to generate electricity which conforms to the fact that summer prevails at this time of the year which results in high irradiance. In Summer, which is around the March–April months, Bangladesh has the highest irradiance values which is 2000 kWh/ $m^{2}$ where it goes down to 1500 kWh/ $m^{2}$ during winter (December-January) which can be seen from Fig. 6 which is obtained from solar PVGIS.

Electricity production in Bangladesh reached 5,008 GWh in February 2021 [28], approximately 40% of which is allocated to Dhaka, which is 2003.2 GWh. According to the data obtained from PVGIS, yearly production of these three lakes using 5% of the total area of the lakes is 19.4 GWh which is about 1% of the overall generation of Dhaka. The coverage area of the plant up to 15%, the generation becomes 3% of the overall generation of the city.

**Fig. 7:**
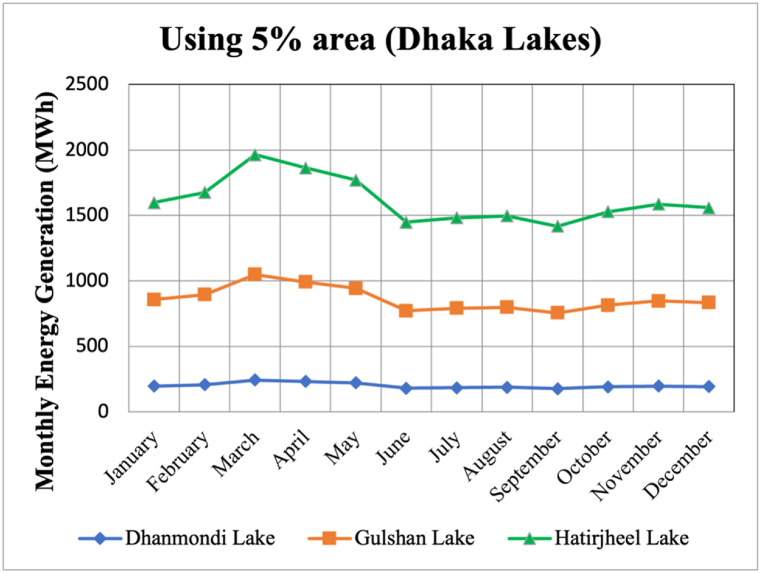
Energy Generation from selected lakes of Dhaka.

Among the three lakes, Hatirjheel lake has the potential to generate the highest amount of electricity and Dhanmondi lake has the least which can be seen from Fig. 7. In addition, from Table 4, the total installed capacity using different areas of the lake can be seen.

**Table 4:** FPV plant capacity of Dhaka lakes.

Lakes	FPV power plant capacity (MW)		
	5% area	10% area	15% area
Dhanmondi	1.7	3.4	5.1
Gulshan	5.7	11.4	17.1
Hatirjheel	6.4	12.9	19.3

#### 3.1.2 Waterbodies outside Dhaka

For assessing the potential for floating PV outside Dhaka city, a few major lakes around the country were considered as prospective sites. In particular, man-made lakes are considered which have large potential for floatovoltaic generation. Those are Joydia Baor (Jhenaidah), Bukbhara Baor (Jessore) and Barapukuria lake (Dinajpur). It is also noteworthy to mention that these lakes were mentioned in feasibility study report, conducted, and published by Asian Development Bank (ADB) to be the potential plants for solar FPV. ADB estimated specific yield to be 1,456 kWh/kWp for Barapukuria, 1,493 kWh/kWp for Bukbhara and 1,495 kWh/kWp for Joydia Baor [15].

**Fig. 8:**
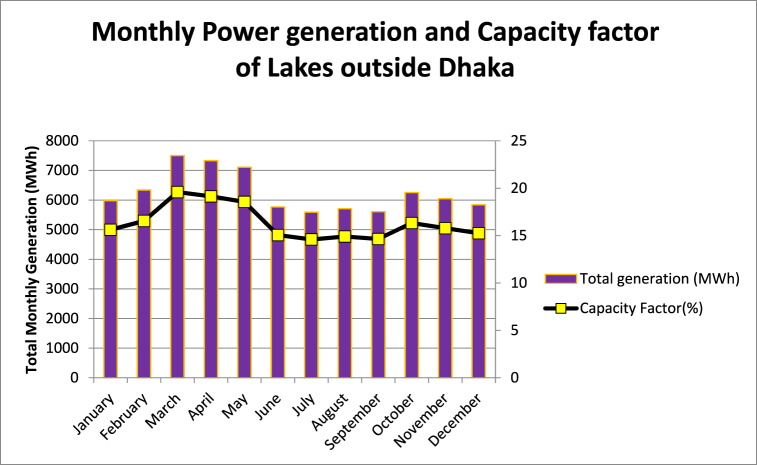
Monthly power generation and capacity factors of FPV installations at selected waterbodies outside Dhaka.

Fig. 8 shows the monthly power generation and the respective capacity factors of the lakes, as found from the study conducted in this paper.

**Fig. 9:**
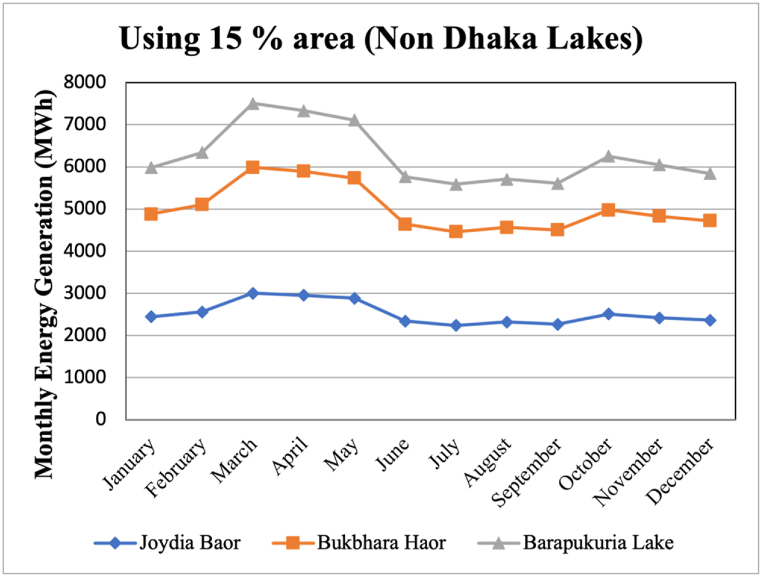
Energy Generation from selected waterbodies outside Dhaka.

The study in this paper took a conservative approach and considered 15% of the total area for floating PV installation. The conservativeness is warranted, considering the human habitation on the banks of the lakes. However, study was also conducted for 20% and 25% of the total area keeping future expansion of the plant in mind. From PVGIS data, it is perceptible that Barapukuria lake possess the highest potential for floatovoltaic generation which is evident in Fig. 9.

The study in this paper conducted specific yield to be 1412.79 kWh/kWp for Barapukuria lake, 1469.243 kWh/kWp for Bukbhara haor and 1470.614 kWh/kWp for Joydia Baor which closely resembles the estimation of ADB. In Table 5, total installed capacity using different areas of the lake can be seen.

**Table 5:** FPV plant capacity of waterbodies outside Dhaka.

Lakes	FPV power plant capacity (MW)		
	15% area	20% area	25% area
Joydia Baor	20.58	27.44	34.30
Bukhbhara Haor	20.44	27.26	34.07
Barapukuria lake	10.46	13.95	17.43

#### 3.1.3 Kaptai Lake

Kaptai lake, located on the South-West of the country, is the largest lake of Bangladesh. It houses the only Hydro-electric power plant (HPP) in Bangladesh. As the total surface area of Kaptai is about 777 sq km [29], it has a huge potential for solar FPV. Therefore, the electricity generated from FPV can take some load off from the HPP and aid to the main grid. Moreover, the water saved from evaporation can be used to increase the HPP generation.

Hybrid operation of the Kaptai HPP and installed FPV can ensure a balanced generation all throughout the year, as confirmed by the results found in this study.

**Fig. 10:**
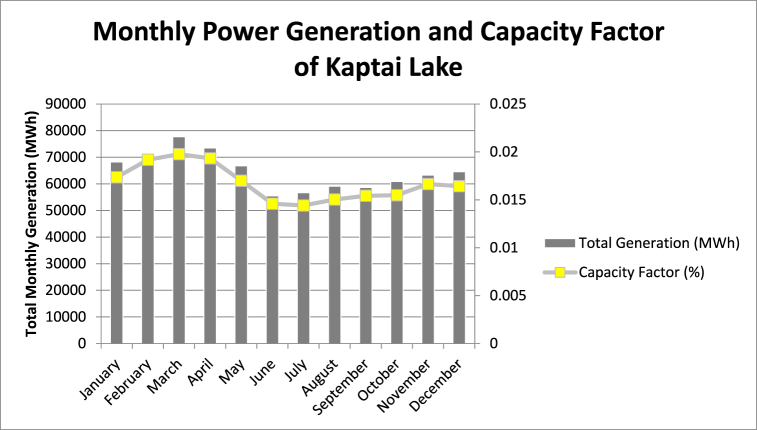
Monthly power generation and capacity factors of FPV installations at Kaptai lake.

To follow a conservative approach 1%, 2% and 3% of the total surface area of the lake was considered for the study. The proposed area can be seen from Fig. 10.

Higher temperature is encountered the summer season than in other time of the year, resulting in greater amount of evaporation. Consequently, the generation capability of HPP reduces drastically during summer. However, installed FPVs could generate the highest amount of electricity during summer. In addition to that, the evaporation loss would be significantly low around the FPV plant due to shading provided by the panels, which eventually aid to the HPP production.

### 3.2 Support to the national grid

#### 3.2.1 Meeting the demand of the capital

One way to understand the potential of floatovoltaic generation in Bangladesh is by assessing how FPVs can aid several load centers around the country. As mentioned before, about 46% of the total demand of the country is attributed to Dhaka, followed by Chattogram as a close second. Owing to such heavy electricity demand at these two load centers, it is investigated how much of the loads at these regions can be met with FPVs, and the results are shown subsequently. Operational reports of Bangladesh power grid were obtained from Power Grid Company of Bangladesh (PGCB) [30].

For calculating average peak demand of Dhaka, twelve (12) representative workdays from each month of the year were chosen. The days were mostly 15th day of the month, unless it was a weekend; in that case nearest weekday was selected. Thereafter, average peak demand was calculated which turned out to be 3847 MW. September had the highest demand with 4636 MW and January had the lowest demand with 3078 MW which can be seen from Table 6 which is collected from BPDB website [23].

**Table 6:** Average peak electricity demand in Dhaka.

Month	Monthly Peak Demand (MW)
January	3078
February	3215
March	3931
April	4189
May	3310
June	4299
July	4465
August	3771
September	4636
October	4554
November	3519
December	3197

The efficiency of the solar panels was assumed to be 17% because most solar panels of the market have efficiency between 15% and 20% [31]. Taking most conservative approach (5% area of the three lakes within Dhaka city),

Total area of the solar panels = 81,430 $m^{2}$.

Therefore, installed peak power = 13.844 MW, which is almost 0.4% of the total demand in Dhaka. However, when 15% area of the lakes are considered,

Total area of the solar panels = 244,310 $m^{2}$.

In that case, installed peak power = 41.53 MW, which is almost 1.1% of the average peak demand in Dhaka.

#### 3.2.2 Meeting the demand of Chattogram circle

The FPV plant established at Kaptai lake can meet a certain portion of the demand of Chattogram city, which encompasses the largest economic zone and seaports of the country.

Twelve (12) days from all the months were selected and average was calculated. The average peak load turned out to be 1198.42 MW. April had the highest demand with 1371 MW and February had the lowest with 899 MW which can be seen from Table 7 [23].

**Table 7:** Average peak electricity demand in Chattogram.

Month	Monthly Peak Demand (MW)
January	1025
February	899
March	1133
April	1371
May	1169
June	1264
July	1319
August	1189
September	1327
October	1337
November	1347
December	1001

In order to maintain a conservative approach (1% surface area of the whole lake), the total generation capability we got from PVGIS turned out to be 88.22 MW, which is more than 7% of the overall demand of Chittagong area.

### 3.3 Economic viability

Up to this point, the potential of the FPV plants was discussed. However, these plants are needed to be economically feasible in order to be deployed practically. In this study, the assessment of economic potential was done based on few parameters which are: LCOE, NPV and IRR. The simulations were done in System Advisory Model (SAM).

#### 3.3.1 Plant design

For the simulations to run, system installed capacity must be given as input. Hatirjheel lake can be taken as an example for better understanding of the process of economic analysis. From technical analysis, it was seen that installed peak power of Hatirjheel was 6.444 MW using 5% of the area of the total lake.

In this study, DC-AC ratio is assumed to be 1.2. Therefore, using Eq. (5), the inverter size should be 5.3MW.

According to the calculations, the selected models of inverter and solar modules are mentioned in Table 8.

**Table 8:** Models of inverter and solar modules.

Device type	Brand name	Model	Specification
Inverter	Delta Electronics	E8-TL-US(AC)	Operating voltage: 240 V
Module	REC Solar	REC255PE-US(BLK)	STC Power Rating: 256.81 W

Similarly, for other the other lakes the inverter size was selected according to the calculations and the modules were taken the same as the one of Hatirjheel analysis. To generate 6.444 MW power, required number of modules with STC power rating of 256.81 W are: 25000modules(approx). In addition, inverter maximum and minimum voltage, and module VOC (Open Circuit voltage) are considered to determine string size. Therefore, using Eqs. (7), (8), maximum and minimum number of modules per string is found to be 13 and 9, respectively. Therefore, 13 modules per string in a subarray were selected which made 1930 strings in parallel in a subarray.

To calculate the installation cost, the module and inverter costs were considered as following based on globally accepted range of values. On top of that, a fixed contingency amount was considered which accounted for the expected uncertainty in the direct cost estimates, which included a percentage of the sum of the equipment, installation labour, and installer margin and overhead costs of the project.

For operating cost, which represents annual expenditures on equipment and services that occur after the system is installed, is considered as a fixed annual cost that is applied to each year in the project cash flow. The assumed values for the calculations can be summarized from Table 9. In a similar way, required calculations were done for the other lakes, by changing the system specifications and keeping the installation and operational costs same as Hatirjheel.

**Table 9:** Assumed values for calculation of economic parameters.

Parameter	Value
Module cost	0.06 USD/Wdc
Inverter cost	0.06 USD/Wdc
Contingency	3%
Fixed cost by capacity	15 USD/kW-yr
Analysis period	25 years
Inflation rate	2.5%/yr
Real discount rate	6.4%/yr
Nominal discount rate	9.06%/yr

#### 3.3.2 Evaluation of economic parameters

From Table 10, NPV is positive in all the cases, which indicates profitability; in other words, the monetary outcome of the project exceeds the installation and operational costs. Next, the LCOE of all the cases are around 4 USD/kWh, whereas the market price of electric energy is 0.053 USD/kWh for households and 0.086 USD/kWh for factories [32]. When LCOE>Marketprice, then project should be implemented. As this is the case for all the proposed water bodies, the projects should be implemented.

**Table 10:** Analysis of economic parameters.

Name of the lake	LCOE ($/kWh)	NPV (x 100000 $)	IRR (%)
Hatirjheel	3.94	2.98	14.92
Dhanmondi	4.86	3.76	11.20
Gulshan	3.41	2.87	18.33
Barapukuria	4.07	3.03	12.56
Bukbhara	3.93	4.77	14.95
Joydia	4.19	3.94	12.17
Kaptai	3.86	292.4	17.10

IRR values came out to be positive and more than 10% which mean the projects would be profitable. Therefore, in all the cases, different economic parameters indicate deployment of FPV plants to be beneficial financially.

## 4 Comparison with inland PV plants

The significant portion of solar based power generation comes from the inland PV plants. For considering FPVs as potential source of power generation, a thorough comparison with inland PV plants is required. To this end, this section conducts a thorough comparison between these two plant types considering energy generation, economic parameters, installation cost and environmental aspects. For inland PV plants, the required information for the comparison purpose is taken from the existing works in the literature. Following that, the calculated results for the proposed FPV plants in this study are used to provide an in-depth comparison. In Table 11, the whole comparison analysis is given with critical insights.

**Table 11:** List of impacts and attributes comparing inland and FPV plants.

Aspect	Inland PV	Floating PV	Comments
Generation capability	Inland PVs generate 4 to 6.5 kWh/m^2^ in daily basis [32].	Floating PVs generate 8.3 kWh/m^2^ in daily basis which is obtained by calculation in this study.	The generation capability clearly indicates FPVs being more efficient which can also be backed up by Ref. [5].
Economic Parameters	IRR of Dhaka, Chittagong and Dinajpur potential inland PV plants are 5.22%, 4.97% and 6,07% respectively [33].	IRR of Dhaka (Dhanmondi, Gulshan and Hatirjheel), Chittagong (Kaptai) and Dinajpur (Barapukuria) FPV plants are 14.82%, 17.10% and 12.56% respectively (Table 7).	IRR suggests FPV plants are profitable by a large margin than the conventional inland PV plants.
Installation Cost	The initial cost of inland PV plants accounts for the solar panels, inverters and wirings.	Besides the panels, inverters and wirings, FPV requires moorings, buoy and support.	Initial installation cost is slightly higher in FPV plants.
Land Acquisition Cost	The land acquisition cost of Dhaka is 85.92 USD/m^2^ [34].	No land acquisition required. Government leasing is sufficient.	FPV costs less than inland ones in this aspect.
Deforestation	Inland PV requires deforestation and usage of cultivable lands.	FPV does not require any form of deforestation.	Inland PV therefore has an adverse impact on the climate change.

## 5 Limitations

This study provides a comprehensive techno-economic assessment of the potential for floating solar systems in selected water bodies of Bangladesh. However, there are certain limitations to this work. These limitations can be used as a steppingstone for improving the FPV research in Bangladesh as well as other South Asian countries with similar irradiation values and climate conditions. The limitations can be mentioned as follows.

• The social aspects of the installation of proposed FPV plants have not been discussed in this study. This includes but not limited to the perception of the general people living in the outskirts of these water bodies and impact on the water body aesthetics. This can be a scope of further research, not only for this specific study, but also throughout the world as studies done on social aspects of FPV are not adequate.
• As this is a case study, it does not include all the water bodies in the country. Further research can be done in order to evaluate the eligibility of other existing water bodies.
• There might be some legal barriers alongside the social ones, such as difficulty to obtain water rights and environmental permits. The study of this legal issues has not been included in this study and can be an opportunity for further research for effective implementation of these proposed plants.

Although the economic parameters show favourable results, the plants may not get implemented because of the limitations mentioned. Nevertheless, the conducted techno-economic analysis can play a significant role in unlocking Bangladesh's floatovoltaic potential with the proper government policies and the removal of social and legal barriers.

## 6 Conclusion

This research conducts a thorough analysis using a proposed methodology to assess the technical and economic feasibility of FPV plants on selected water bodies in Bangladesh. In addition, this research is the first to analyze the contribution of FPVs to the national grid. Furthermore, a comparison of the planned FPV plants with their inland counterparts is performed, considering several aspects. Even when geographical variables and system losses are taken into account, the proposed plants indicate strong generating potential in each of the case studies. Intriguingly, the findings indicate that the proposed plants in Dhaka could meet 1.1% of the city's entire demand, and that those in Kaptai Lake could satisfy 7% of Chattogram, the country's port city. In terms of the economic evaluation (LCOE, NPV, and IRR), each of the proposed plants demonstrates project viability following implementation. Although the proposed FPV plants initially cost more than inland PV plants, the projected plants have superior generating capabilities and profitability than the inland counterparts.

However, further study is required to assess the social and legal barriers that these FPV installations may confront. The study may be expanded further by exploring the Feed in Tariff (FIT) approach that several countries, including Vietnam, utilize for their FPV plants [35]. Moreover, the water conservation benefits of implementing these plants may be a promising focus for future research. As this is the first technical and economic analysis of the potential of FPV plants on several important water reservoirs of Bangladesh, it will certainly open doors for further research and analysis. Through large-scale implementation of FPV plants, Bangladesh can significantly reduce the energy crisis and generate adequate power for meeting the local demand.

## Author contribution statement

Md. Fatin Ishraq Faruqui, Atik Jawad: Conceived and designed the experiments; Performed the experiments; Analyzed and interpreted the data; Contributed reagents, materials, analysis tools or data; Wrote the paper.

Nahid-Al-Masood: Analyzed and interpreted the data; Contributed reagents, materials, analysis tools or data; Wrote the paper.

## Data availability statement

Data will be made available on request.

## Declaration of competing interest

The authors declare that they have no known competing financial interests or personal relationships that could have appeared to influence the work reported in this paper.

## Figures and Tables

**Fig. A.1:**
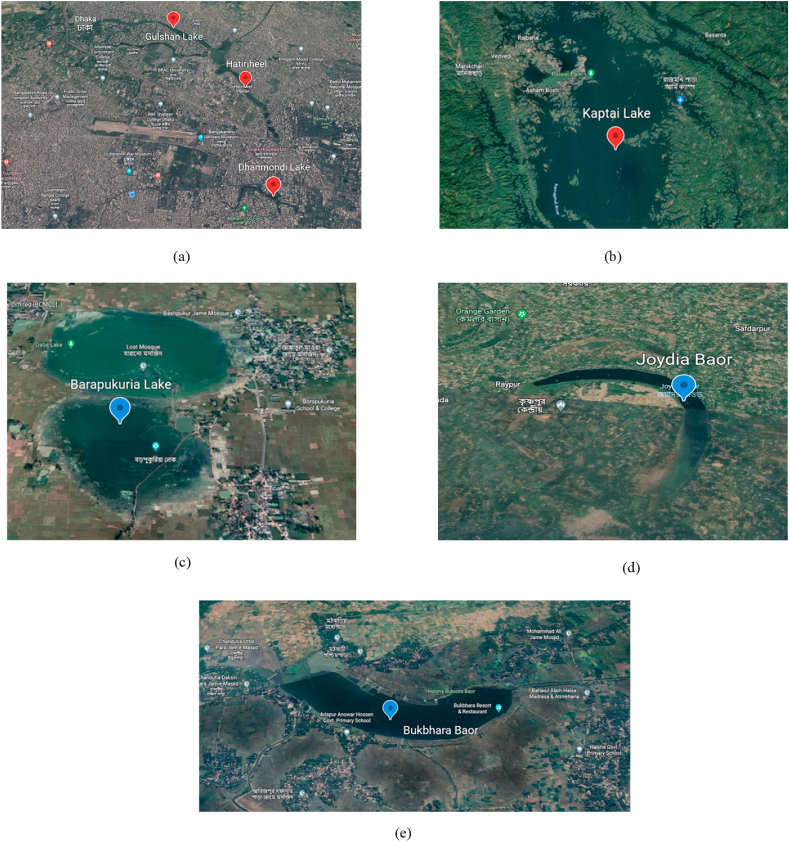
Geographical location of the proposed sites (a) Lakes of Dhaka (Dhanmondi, Gulshan and Hatirjheel lakes), (b) Kaptai lake, (c) Barapukuria lake, (d) Joydia Baor, and (e) Bukbhara Baor

**Table A.1:** Latitude and Longitude of the proposed sites

Lake	District	Latitude	Longitude
Dhanmondi Lake	Dhaka	23.744151	90.3777709
Hatirjheel Lake	Dhaka	23.758088	90.404818
Gulshan Lake	Dhaka	23.776822	90.419624
Barapukuria Lake	Dinajpur	25.538594	88.968640
Bukbhara Baor	Jessore	23.173346	89.114927
Joydia Baor	Jhenaidah	23.442163	88.946493
Kaptai Lake	Rangamati	22.602119	92.199487

**Fig. A.2:**
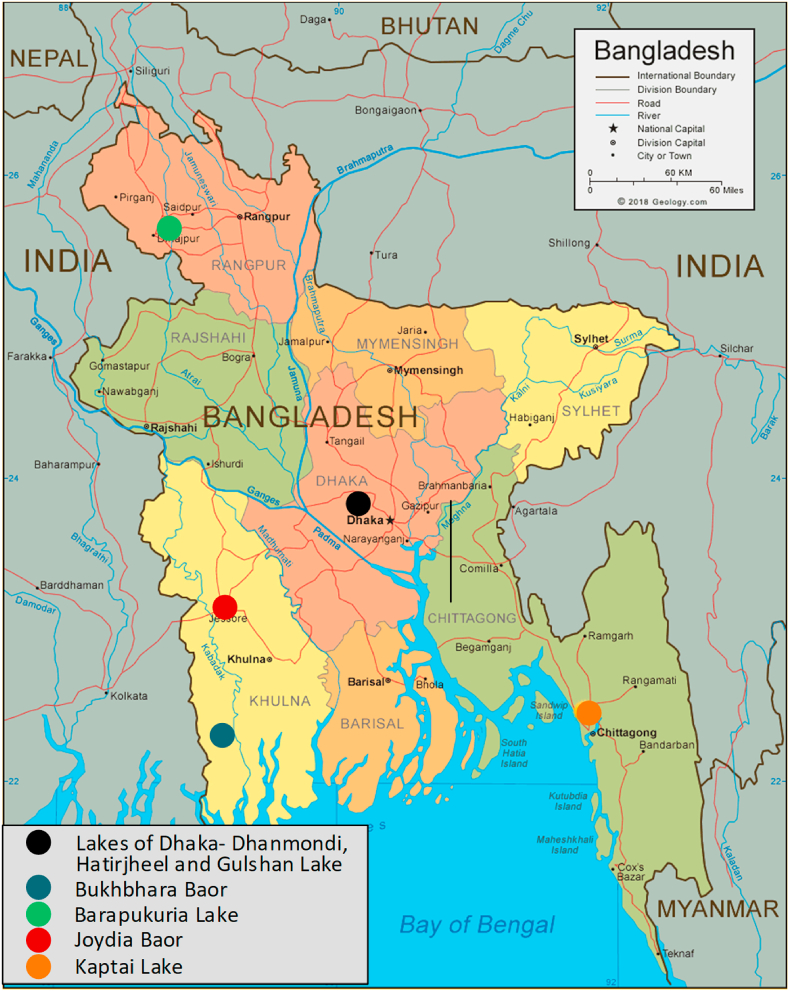
Map of Bangladesh with the proposed sites marked.

## Appendix

Geographical Location of the Proposed Lakes.
